# Patient activation is a treatable trait in patients with chronic airway diseases: An observational study

**DOI:** 10.3389/fpsyg.2022.947402

**Published:** 2022-10-05

**Authors:** Jeannette B. Peters, Jeanine C. Antons, Eleonore H. Koolen, Hanneke A. C. van Helvoort, Hieronymus W. H. van Hees, Bram van den Borst, Martijn A. Spruit, Jan H. Vercoulen, Alex J. van ’t Hul

**Affiliations:** ^1^Department of Pulmonary Diseases, Radboud Institute for Health Sciences, Radboud University Medical Center, Nijmegen, Netherlands; ^2^Department of Pulmonary Diseases, Donders Institute for Medical Neuroscience, Radboud University Medical Center, Nijmegen, Netherlands; ^3^Department of Research and Development, CIRO, Horn, Netherlands; ^4^Department of Respiratory Medicine, Maastricht University Medical Center (MUMC), Maastricht, Netherlands; ^5^Faculty of Health, Medicine and Life Sciences, NUTRIM School of Nutrition and Translational Research in Metabolism, Maastricht University, Maastricht, Netherlands; ^6^Department of Medical Psychology, Radboud Institute for Health Sciences, Radboud University Medical Center, Nijmegen, Netherlands

**Keywords:** self-management, pulmonary disease, decision making, consultation, motivation, COPD, asthma

## Abstract

**Background:**

Self-management is key for reducing the burden of disease in chronical illness. However, applying self-management presupposes behavioral change. Sufficient knowledge, skills, confidence and motivation to make the needed behavior changes are important prerequisites. During the past years the Integral Diagnostic Trajectory was developed for patients with asthma or COPD which aims to identify treatable traits and activating patients for self-management.

**Objective:**

In the present study the effects of the Integral Diagnostic Trajectory on the Patient Activation Measure (PAM^®^) were examined. In addition, predictive variables for PAM baseline scores and change scores were sought.

**Materials and methods:**

A total of 241 patients with asthma or COPD referred to the pulmonologist at the Radboud university medical center, location Dekkerswald, Nijmegen were included. Patient activation was measured before the first visit and after the intervention with the 13-item PAM^®^. Additional, patient characteristics and health status were measured with the Nijmegen Clinical Screening Instrument (NCSI), modified Medical Research Council (mMRC), Asthma Control Questionnaire (ACQ), and COPD Clinical Questionnaire (CCQ).

**Results:**

Fifty percent of the patients with asthma and seventy percent of the patients with COPD had low levels of activation at baseline (PAM level 1–2). Baseline PAM scores could be explained in patients with asthma for 7% by number of severe problems in health status. And for 18% in patients with COPD by number of severe problems, age and employment status. After the intervention both groups significantly improved on the PAM (T_0_: 56.0 ± 13.1 vs. T_1_:63.3 ± 14.0 in asthma, and T_0_: 50.0 ± 8.8 vs. 58.4 ± 11.1 in COPD). Multivariate stepwise regression analysis showed that only 24% of the change in score could be explained by baseline PAM score and being employed in patients with asthma, and 18% of the variance in change score could be predicted by baseline PAM score in COPD.

**Conclusion:**

The present study showed that low level of activation is a common feature in patients with asthma and COPD. With a relatively short and seemingly simple intervention patients can reach higher levels of patient activation, which is a prerequisite for adopting self-management techniques in daily life.

## Introduction

Adequate self-management of one’s health is deemed a crucial issue in patients’ chronic conditions to reduce its burden for both the individual and for society (Allegrante et al., 2019). For instance, patients with chronic conditions and poor self-management are more often subjected to emergency room visits, to be hospitalized and/or to be readmitted (Barker et al., 2018). Also for patients with asthma and chronic obstructive pulmonary disease (COPD), improving self-management has been acknowledged as key component of individual disease management (Pinnock et al., 2015; Effing et al., 2016). An international group consensus defined a self-management intervention as structured but personalized and often multi-component, with goals of motivating, engaging, and supporting the patient to positively adapt their health behaviors and develop skills to better manage their disease (Effing et al., 2016). The ultimate goals of self-management are: (a) optimizing and preserving physical health; (b) reducing symptoms and functional impairments in daily life and increasing emotional wellbeing, social wellbeing, and quality of life (QoL); and (c) establishing effective alliances with healthcare professionals, family, friends, and community (Effing et al., 2016).

To successfully take on the challenges that lie within applying self-management in daily life, that is, making the required behavioral adaptations, people need knowledge, skills, and confidence. This is defined as the level of activation for self-management, which can be measured with the Patient Activation Measure (PAM^®^) (Hibbard et al., 2004; Rademakers et al., 2012). Indeed, studies have shown that patients with COPD with higher levels of activation have a better ability to perform self-management activities (Greene and Hibbard, 2012), have a lower likelihood of having a severely impaired health status (van ‘t Hul et al., 2020), better health outcomes (Hibbard and Greene, 2013), better health care experiences (Hibbard and Greene, 2013), better knowledge and self-efficacy (Chang and Dai, 2019), more motivation to persist in exercise (Nguyen et al., 2009), better mood (Titova et al., 2017), and show lower healthcare utilization (Titova et al., 2017).

Unfortunately, several cross-sectional studies in primary and secondary care settings have shown that PAM scores are generally low in patients with chronic respiratory conditions, showing percentages of 50–75% of patients with a PAM level 1 or 2, meaning they are not aware of their problems and their own role or miss knowledge and self-esteem to embrace the need for treatment (Bos-Touwen et al., 2015; Korpershoek et al., 2016; Collinsworth et al., 2018; Janssen et al., 2020; Bloem et al., 2022). Clearly there is a need to improve patient activation in usual care. Moreover, there is emerging evidence that personalized interventions support building better skills and confidence and are effective in increasing patient activation (Hibbard and Greene, 2013). Ideally, both the patient as well as the healthcare provider have to take a role and share the responsibility for improving activation levels and adopting self-management techniques. However, studies applying and evaluating patient activation are scarce (Yadav et al., 2018). During the past years, the COPDnet integrated care model was developed for patients with chronic airway diseases and a series of studies was conducted to objectify the added value on change in health status (Koolen et al., 2018b,^2020^). The COPDnet integrated care model consist of an Integral Diagnostic Trajectory in a secondary care setting, which is followed by non-pharmacological interventions in primary or secondary care based on the individual care plan that was composed during the integral diagnostic trajectory (Koolen et al., 2018a). In the present study, we will focus on the first part, the Integral Diagnostic Trajectory. The Integral Diagnostic Trajectory aims at making a comprehensive analysis of patients’ overall health status, and simultaneously increase the patients’ level of activation for self-management. We will examine the effect of the Integral Diagnostic Trajectory on PAM scores. In addition, predictive variables were sought for baseline PAM scores as well as changes that occurred at completion of the Integral Diagnostic Trajectory.

## Materials and methods

### Research design and participants

This observational study was conducted with data from 241 patients with asthma or COPD who were referred to the pulmonologist at the Radboud university medical center, location Dekkerswald, Nijmegen, the Netherlands. In this study all patients were included for whom this was the first-time referral to the pulmonologist, with a confirmed diagnosis of asthma or COPD and who completed the Integral Diagnostic Trajectory between July 2016 and January 2020. Patients were excluded from this study for the following reasons: those who had an acute exacerbation in the past 3 months prior to referral, who were unable to complete the questionnaires due to cognitive impairment or who were unable to speak or understand the Dutch language.

### Ethical considerations

The study was conducted in accordance with European Union directive 2001/20/EC and the Declaration of Helsinki. The Research Ethics Committee of the Radboud University Medical Centre reviewed and approved the study and considered that the study protocol did not fall within the remit of the Medical Research Involving Human Subjects Act (WMO) (ref: 2017/3597). As this study had an observational nature all measurements were obtained as part of usual care. No additional measurements were needed for this study. Data was anonymized before analysis. Baseline results have been presented before at the European Respiratory Society annual congress (Koolen et al., 2017).

### Measures

To measure patient activation level the Patient Activation Measure-13 (PAM^®^) (Hibbard et al., 2005; Rademakers et al., 2012) was assessed before the first visit (T_0_) and directly after visit two or three (T_1_). The PAM^®^ consists of 13 items with response categories on a 4-point Likert scales ranging from “totally disagree” to “totally agree,” and “non-applicable.” The PAM^®^ appears to be a valid and reliable instrument to measure activation (Hibbard et al., 2005). The study by Hibbard et al. (2005) shows that the Cronbach’s coefficient α was 0.91 in a group of 486 respondents, of which 120 cardiac rehabilitation patients and 366 employees in a large health system in second community, of whom in total 76% reported having a chronic condition. In the current study the Cronbach’s coefficient α is 0.67 at T_0_ and 0.82 at T_1_. With a calculation tool, provided by Insignia Health, raw data was transformed to a standardized patient activation score ranging from 0 to 100. Higher scores indicate greater activation. Based on the score patients were assigned into one of four activation levels: level one (PAM score ≤ 47) “people tend to be overwhelmed and unprepared to play an active role, and are considered passive recipients of care,” level 2 (PAM score 47.1-55.1) “individuals lack knowledge and confidence for self-management,” level 3 (PAM score 55.2-67.0) “people are beginning to take action, but still may lack confidence and skills to support new behavior,” level 4 (PAM score ≥ 67.1) “people have confidence and perform adequate behavior, but may not be able to maintain them in the face of stress.” An improvement in 4 points on the PAM scale is considered a minimal clinically important difference (MCID). Permission for using the PAM^®^ was obtained from Insignia Health.

**FIGURE 1:**
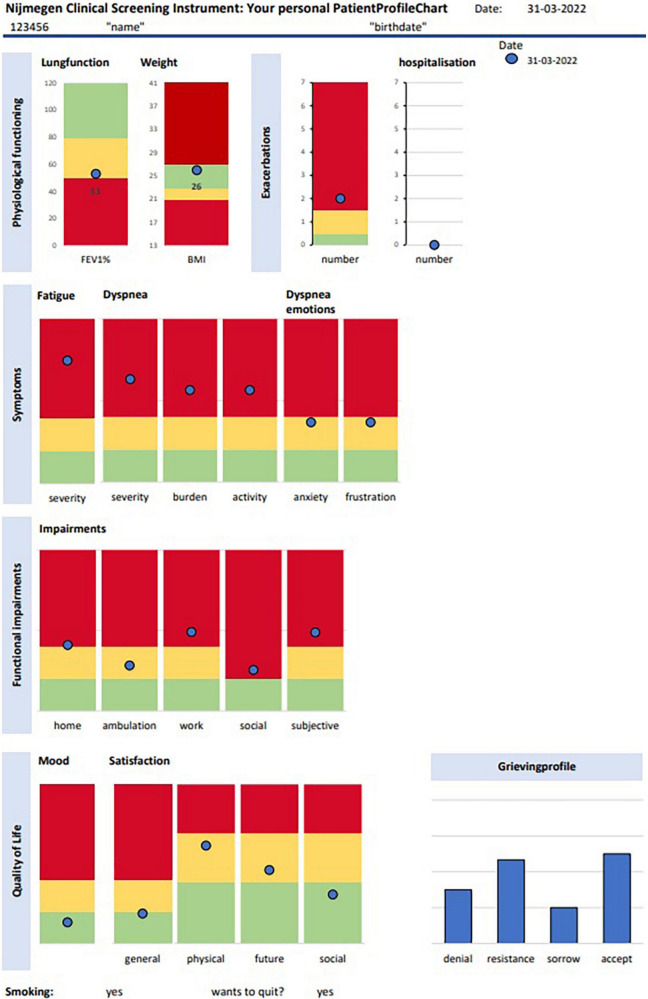
Patient profile chart. The green score range indicates “normal functioning,” the yellow score range indicates “mild problems,” and the red score range indicates “severe problems.” Accep, acceptance.

Health status was measured with the Nijmegen Clinical Screening Instrument (NCSI) before the first visit (T_0_). (NCSI) (Peters et al., 2009). The NCSI is a valid and evidence-based battery of different instruments to measure many aspects of health status with as little items as possible. The NCSI covers three domains of integral health status: Symptoms, Functional impairment and QoL, which each are subdivided into several sub-domains. The main domain symptoms is subdivided into the three subdomains dyspnea, dyspnea- related emotions and fatigue and measures the overall burden of pulmonary symptoms (Vercoulen et al., 2008), the level of frustration and anxiety a person experiences when dyspnoeic (Vercoulen et al., 2008), and the level of experienced fatigue (Vercoulen et al., 1994). The Cronbach’s α in this study are, respectively 0.86, 0.87, and 0.90. The domain functional impairments consist of the two sub-domains behavioral impairment and subjective impairment that measure the extent to which a person cannot perform specific and concrete activities as a result of having the disease (Bergner et al., 1981) and the experienced degree of impairment in general (Maille et al., 1997). The Cronbach’s α for these two subdomains are, respectively 0.78 and 0.88 in this study. The main domain QoL consists of three sub-domains general QoL, health related QoL, and satisfaction with relations and measures mood and satisfaction of a person with his/her life (Diener et al., 1985; Beck et al., 1997), the satisfaction related to physical function and the future (Vercoulen et al., 2008) the satisfaction with the (absent) relationships with spouse and others (Vercoulen et al., 2008). The Cronbach’s α in this study are, respectively 0.60, 0.63, and 0.69. For all sub-domains the higher the score the more problematic. Cut-offs are available indicating normal functioning (comparable with healthy persons), mild problems or severe problems for each of the sub-scales. The NCSI was completed by the web-based software RadQuest. Results are automatically transformed into the graphical *PatientProfileChart* (PPC) (see Figure 1). Lung function, number of exacerbations and body mass index are also displayed on the PatientProfileChart (PPC). In addition, the stages of the grieving process (denial, resistance, sorrow, acceptance) regarding the chronic illness were measured and displayed on the PPC (Boer et al., 2014).

Demographics were collected at first visit (T_0_). These include age, gender, BMI, pulmonary function, the modified Medical Research Council (mMRC) (Mahler and Wells, 1988), employment status, and education level according to Verhage’s classification (Verhage, 1965). In patients with COPD also the COPD Clinical Questionnaire (CCQ) was measured to measure the degree of disease burden (van der Molen et al., 2003). The Cronbach α in this study is 0.86. A cut-off of 1.9 points was used to discriminate between low or high disease burden. In patients with asthma the level of asthma control was measured with the Asthma Control Questionnaire (ACQ) (Juniper et al., 1999). The Cronbach α in this study is 0.84. Scores below 0.75 points indicate the asthma is under control, scores between 0.75 and 1.5 partially controlled and score above 1.5 indicate uncontrolled asthma.

### Integral diagnostic trajectory—Intervention

Briefly, the intervention consisted of two to three visits, with exactly 1 week between the first and second visit. On indication a third visit was scheduled 3–6 weeks after the second visit. Before the first visit patients were asked to fill in the PAM at home.

During the first visit assessments were performed to get a comprehensive picture of the patients’ overall health status (van ‘t Hul et al., 2020). Symptoms, Functional Impairment and QoL were assessed using the NCSI (Peters et al., 2009). At the same visit, the pulmonologist and specialized respiratory nurse consulted with the patients. During these consultations they focused on the biomedical aspects, including optimizing and adherence to pharmacotherapy, smoking, activity behavior, nutritional status, mood, coping with the disease, and self-management behaviors. During the consultations, besides gathering and providing information, most important was establishing a working relation between the patient and clinician. Main goals of visit one: to confirm the medical diagnosis, identify the level of activation and identify the number and complexity of the problems in health status (van ‘t Hul et al., 2020).

During the second visit the results of all assessments from visit 1, gathered from the performed tests, activity monitor, questionnaires and consultations, were discussed with the patient. Main goal of the consultations (and if necessary, a third consultation) is to construct an individual care plan together with the patient for optimizing the patients’ health status that fits their own needs, capabilities and desires. To come to this, several steps had to be taken. During the appointment with the pulmonologist among other things the diagnosis and other medical issues were discussed with the patient. During this discussion the pulmonologist checked for gaps in the patients’ knowledge, his/her attitude toward treatment options, provided information to fill the knowledge gap or correct irrational/unhelpful ideas, and discussed possible treatment options in order to optimize the biomedical aspects. The respiratory nurse discussed the PPC in a semi-structured way with patient and partner/spouse. In fact, this specific intervention entails Motivational Interviewing, but is greatly aided by the semi-structured discussion of the PPC. A detailed description of the NCSI-intervention and motivational processes involved can be found elsewhere (Vercoulen, 2012). This intervention has several effects which increase patient activation: (1) The patient becomes aware of the severity of his health status problems; (2) Resistance is easily dealt with as the patient has completed the questionnaire himself and the visual presentation has far more impact than mere words; (3) The patient becomes aware of the role of his own behavior in the health status problems seen on the PPC; (4) Not only the patient and caregiver get these insights, but also the partner.

The objective of the second visit is to let the patient formulate treatment goals and to establish an individual care plan for optimizing the patients’ health status. The individual care plan is made together with the specialized respiratory nurse. In some patients the individual care plan was finalized in the second consultation. For other patients a third visit was required, to give the patient time to think about all what had been discussed, more time to form their own opinion or to discuss it with their spouse or other important persons.

### Statistical analysis

Due to the retrospective nature, no formal *a priori* sample size calculation was performed.

Patients who completed the PAM before the first visit (T_0_) were included in this study. Baseline descriptions are presented as mean ± standard deviation for continuous variables and number and percentages for categorical data. Differences between baseline characteristics of patients with asthma or COPD were tested with an independent samples *t*-test for continuous variables and using the Chi-square test to compare proportions. A multiple linear regression analysis with a stepwise method was performed to identify variables from the baseline characteristics associated with the PAM score at T_0_. PAM score at T_0_ was used as dependent variable and sex, age, FEV_1_% of predicted, education level, employment status, mMRC and number of severe problems on NCSI as independent variables. The analyses are performed for both patient groups separately if PAM scores at T_0_ would differ significantly between the two patient groups.

To test changes in PAM scores after the intervention a *t*-test was performed on the PAM scores at T_0_ and T_1_. Individual responses on the PAM score were calculated at T_1_ subtracting the baseline score (T_0_) from the score after the intervention (T_1)_. Responses were dichotomized applying the clinically meaningful change definition of 4 points and the fraction of positive responders was determined (Hibbard et al., 2007). A multiple linear regression analysis with a stepwise method was performed to identify variables from the baseline characteristics associated with change in PAM scores (Delta PAM). For this, the PAM change score from T_0_ to T_1_ was used as dependent variable. The following independent variables were considered: age, gender, FEV_1_% of predicted, mMRC, smoking, education, employment status, number of severe problems on NCSI, PAM score at T_0_, number of visits (two or more than two). SPSS version 25 (IBM, Chicago, IL, USA) was used to run the statistical analysis and a *P*-value of < 0.01 was considered statistically significant.

## Results

### Patient characteristics

Baseline characteristics of 241 patients with asthma or COPD of who met de inclusion criteria are presented in Table 1. All patient characteristics, except sex, differed significantly between patients with asthma or COPD. In one third of the patients the individual care plan was completed in two visits, no significant differences were found on PAM T_0_ between patients with two consultation days and patients with three consultation days (T_0_: 54.7 ± 13.0 vs. 53.0 ± 10.8; *p* > 0.05). Fifty percent of patients with asthma and sixty percent of patients with COPD had low levels of activation at baseline. Patients with COPD had significantly lower scores on the PAM at T_0_, and reported significantly more subdomains with severe problems as compared to patients with asthma. On all subdomains of the NCSI proportions of patients with severe problems ranging from 17 to 71% were found in both patient groups, with highest percentages in fatigue, dypnea emotions, and QoL. Of all NCSI-domains, only behavioral impairment and subjective dyspnea were significantly different between both groups. High symptom burden was found in forty percent of patients with COPD as measured with the CCQ and uncontrolled asthma was found in half of the patients with asthma, measured with the ACQ.

**TABLE 1:** Patient characteristics and percentages of severe problems on NCSI, CCQ and ACQ at baseline for whole study group and for confirmed diagnosis of asthma and COPD.

	Total (*N* = 241)	Asthma (*n* = 134)	COPD (*n* = 107)	*P*-value
Age, years	55.6 ± 15.1	48.8 ± 15.5	64.0 ± 9.1	<0.01
Sex, male	39% (94)	37% (49)	42% (45)	n.s.
Employed	50% (117)	65% (86)	30% (31)	<0.01
Educational level				<0.01
Low	14% (29)	6% (7)	22% (21)	
Medium	46% (98)	41% (48)	53% (50)	
High	40% (86)	54% (63)	24% (23)	
Smoking, yes	20% (47)	11% (15)	30% (32)	<0.01
VCmax% of predicted	95.7 ± 15.9	98.4 ± 13.6	92.4 ± 17.9	<0.01
FEV_1_% of predicted	73.8 ± 23.9	83.5 ± 18.9	61.6 ± 23.9	<0.01
FEV_1_/VC ratio	76.2 ± 20.4	84.8 ± 17.0	65.5 ± 19.2	<0.01
BMI, kg/m^2^	27.0 ± 5.9	27.2 ± 5.4	26.7 ± 6.5	n.s.
PAM mean score, points	53.6 ± 11.6	56.3 ± 12.9	50.3 ± 8.9	<0.01
Level 1	37% (89)	28% (38)	48% (51)	<0.01
Level 2	21% (51)	22% (29)	21% (22)	
Level 3	35% (84)	40% (53)	29% (31)	
Level 4	7% (17)	10% (14)	3% (3)	
Number of visits > 2	65% (157)	64% (86)	66% (71)	n.s.
NCSI QoL, severe	56% (118)	51% (61)	61% (57)	n.s.
NCSI HrQoL, severe	33% (70)	31% (37)	36% (33)	n.s.
NCSI Sat. relations, severe	16% (33)	14% (17)	17% (16)	n.s.
NCSI Subj. impairment, severe	53% (113)	51% (61)	56% (52)	n.s.
NCSI Beh. impairments, severe	38% (81)	31% (26)	54% (50)	<0.01
NCSI Subj dyspnea, severe	53% (112)	45% (53)	63% (59)	<0.01
NCSI dyspnea emotions, severe	57% (121)	56% (66)	59% (55)	n.s.
NCSI fatigue, severe	63% (134)	57% (68)	71% (66)	n.s.
NCSI, no subdomains with severe problems	3.24 ± 2.51	2.94 ± 2.46	3.62 ± 2.53	<0.01
mMRC score	1.19 ± 1.19	0.86 ± 1.03	1.60 ± 1.26	<0.01
CCQ score*			2.04 ± 1.09	
CCQ high symptom burden*			40% (43)	
ACQ score*		1.62 ± 1.05		
ACQ controlled asthma*		19% (19)		
ACQ partially controlled*		31% (32)		
ACQ uncontrolled *		50% (51)		

*29 patients did not complete all questionnaires, and due to incorrect diagnosis at referral data was missing from 14 patients with COPD on the CCQ and from 32 patients with asthma on the ACQ. PAM, Patient Activation Measure; FEV_1_, forced expiratory volume; VC, vital capacity; BMI, Body Mass Index; NCSI, Nijmegen Clinical Screening instrument; QoL, Quality of Life; HrQoL, Health related quality of life; Sat, satisfaction; Subj, subjective; Beh, behavioral; mMRC, modified Medical Research Council; CCQ, Clinical COPD Questionnaire; ACQ, Asthma Control Questionnaire; *p*-value of either *t*-test or chi-square depending on variable.

A multiple linear regression analysis with PAM score at T_0_ as dependent variable in patients with asthma showed that 7% of the variance in PAM T_0_ score could be explained by number of severe problems, whereas gender, age, FEV_1_% of predicted, education level, employment status, and mMRC did not contribute to the variance (*p* < 0.001). In patients with COPD 18% of the variance in PAM T_0_ score could be explained by number of severe problems, age and employment status. Whereas gender, FEV_1_% of predicted, education level, and mMRC did not contribute to the variance (*p* < 0.001).

### Patient activation measure scores over time

**FIGURE 2:**
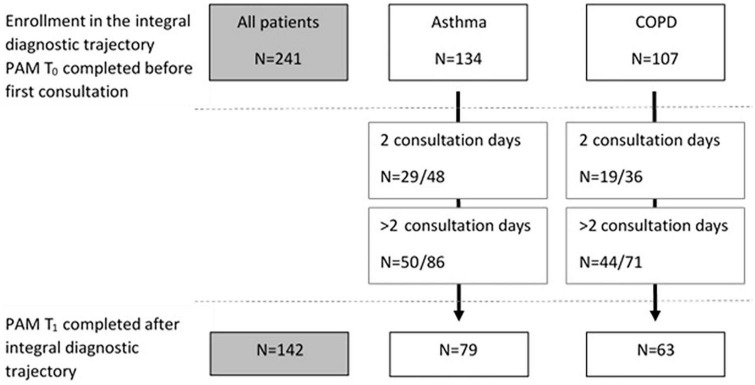
Flowchart of patients who completed the PAM at baseline (T_0_) and number of patients who completed the PAM after the intervention (T_1_), for the whole group and for asthma and COPD separately. For both groups the numbers of patients who completed T_1_ of those with two consultations and number of patients who completed T_1_ of those with more than two consultations are included.

Seventy-nine (59%) patients with asthma and 63 (59%) of the patients with COPD completed both the PAM before and after the intervention (Figure 2). In both patient groups no significant differences on PAM T_0_ were found between patients who completed and who did not complete the PAM at T_1_ (asthma mean PAM score at T_0_: 56.7 ± 12.6 vs. 56.0 ± 13.1, *p* = 0.77, and COPD mean PAM score at T_0_: 50.6 ± 9.2 vs. 50.0 ± 8.8, *p* = 0.73, respectively).

**FIGURE 3:**
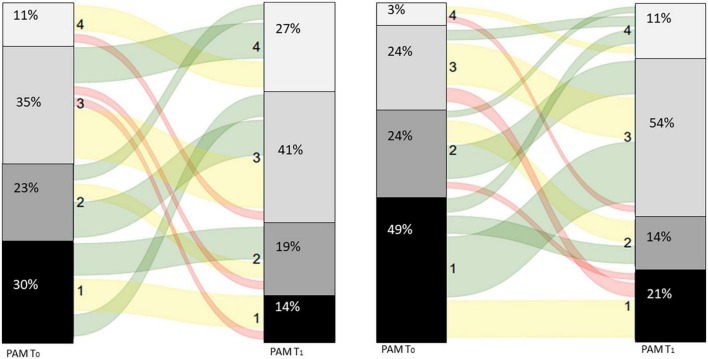
Sankey diagram for patients with asthma **(left panel)** and patients with COPD **(right panel)**.

At first visit (T_0_) half of the patients with asthma, who completed both PAM questionnaires, reported low levels of activation (PAM level 1–2, Figure 3). There was a significant improvement between the scores on T_0_ (T_0_: 56.0 ± 13.1) and T_1_ (63.3 ± 14.0) in patients with asthma, *p* < 0.01 (Table 2). Consequently, the proportion of patients with asthma with PAM level 1 or 2 reduced to one third.

**TABLE 2:** PAM scores at baseline (T_0_) and after the intervention (T_1_) for patients whom completed both measurements.

	Asthma (*N* = 79)		COPD (*N* = 63)	
	T0	T1	T0	T1
PAM score	56.0 ± 13.1	63.3 ± 14.0*	50.0 ± 8.8	58.4 ± 11.1*
Delta PAM T_0_-T_1_	7.2 ± 10.6		8.3 ± 11.7	
Delta PAM T_0_-T_1_ > MCID	66% (52)		65% (41)	

PAM, patient activation measure; MCID, Minimal clinical important difference. *paired *t*-test, *p* < 0.01.

Seventy-three percent of the patients with COPD reported low levels of activation, their belief in own role and confidence levels are low before the first visit (T_0_, Figure 2). There was a significant improvement between the scores on T_0_ (T_0_: 50.0 ± 8.8) and T_1_ (58.4 ± 11.1) in patients with COPD, *p* < 0.01 (Table 2). Resulting in a decrease in patients with COPD to one third with scores belonging to PAM levels 1 or 2.

### Predictors of change in patient activation measure score

Multiple linear regression analysis showed that baseline PAM score and employment explained 24% of the variance in change in PAM score between T_0_ and T_1_ (PAM delta = 18.032–0.285*PAM score T_0_ + 8.039 in case of employment *p* = 0.003) in patients with asthma.

In patients with COPD only the PAM score at T_0_ explained 18% of the variance in change score (PAM delta = 35.174–0.545 *PAM score T_0_, *p* = 0.001).

## Discussion

In the present study we found high percentages of patients with asthma or COPD with low activation levels at baseline who were referred to the Integral Diagnostic Trajectory. Baseline activation scores could only be predicted for 7%, by the number of severe problems, in patients with asthma, and for 18% in patients with COPD, by number of severe problems, age and employment status. In two third of the patients, we found a clinically relevant improvement in the PAM-score after two or three visits. The baseline PAM score and being employed could explain 24% of the variance in change scores in patients with asthma, and baseline score alone could explain variance in change scores for 18% in patients with COPD, leaving much room for other contributing factors. The results show that the COPDnet Integral Diagnostic Trajectory can have a positive effect on the activation level of patients with asthma and COPD.

We found high percentages of patients with low activation levels at baseline in patients with asthma or COPD referred to the pulmonologist. Moreover, the patients with COPD in the present study had significantly lower activation scores compared to patients with asthma and compared to mean scores of other studies in patients with COPD (Turner et al., 2014; Bos-Touwen et al., 2015; Korpershoek et al., 2016; Titova et al., 2017). Furthermore, the activation levels of both groups were considerably lower than found in other chronic diseases at baseline (Rademakers et al., 2012; Rygg et al., 2012; Titova et al., 2017; Humphries et al., 2021). Hence, despite all care that has been provided by the general practitioner, or other professionals in primary care, these patients still seem to miss the required self-management skills and knowledge for reducing their disease burden. Having a chronic (progressive) disease affects daily life and requires adaptation of behavior by including self-management techniques such as inhalation techniques, medication adherence, energy saving techniques, breathing regulation, continuing to exercise, or apply stress-management (Effing et al., 2016). However, self-management techniques are obviously not automatically adopted. One has to be motivated, engaged, and feel supported to adapt the new desired behavior and to develop skills to better manage their disease (Effing et al., 2012). Moreover, the patient should have sufficient knowledge, skills and be confident that they can perform these behaviors adequately. The high percentages of patients with low activation levels at baseline, indicate that more than half of the patients in this study generally lack knowledge and skills about their disease and the importance of one’s own role in disease management.

Increasing the patients’ level of activation for self-management is central in the present care model of the hospital-based outpatient Integral Diagnostic Trajectory to improve the patients’ overall health status (Koolen et al., 2018a). Patients are enticed to play an active role in the consultations with the pulmonologist and specialized respiratory nurse. Together they discuss the results of the various tests, their medical diagnosis, and other aspects of the patients’ health status. In addition, by increasing the patients’ awareness of the role of their own behavior regarding their health problems the patient understand that they have to change behavior and adopt adequate self-management behaviors to improve their health status. Treatment goals and interventions then logically follow from these insights. During the consultations the pulmonologist and the specialized respiratory nurse apply the key components of motivational interviewing (Miller and Rollnick, 2002) and shared decision making (Elwyn et al., 2017). By forming a team, active listening, checking whether the patient understands all, has questions or concerns, and asking which goals are most important for the patient. All to increase the patients’ motivation to take an active role in making an individualized care plan that involves improvement and adaptation of self-management in daily life.

Several intervention studies in patients with COPD aiming at improving self- management and patient activation found significant improvements on patient activation directly after the intervention (Deen et al., 2011) and after 3–6 months (Nguyen et al., 2009; Titova et al., 2017; Collinsworth et al., 2018; Chang and Dai, 2019) whereas other studies showed lack of improvement on patient activation after the intervention (Eikelenboom et al., 2016; North et al., 2020). The differences between the studies with a positive effect on patient activation and those without seem to be due to whether the intervention was performed as intended. Eikelenboom et al. (2016) for example argued that possible explanations could be that firstly, the general practitioners (GP) were not familiar enough with the techniques they had to use, training was not sufficient, secondly, the GPs sometimes forgot to discuss the results, results were not available at the consultation, or an extra consultation was needed. In contrast to, as far as we know, the health care professionals in the other studies all followed the study protocol, felt comfortable in their role, and every patient was offered sessions to enhance knowledge and making a self-management plan. Lack of self-efficacy, knowledge, and experience are known barriers for using shared decision making by clinicians (Driever et al., 2020; Koyama et al., 2022). Although, most clinicians would prefer using shared decision making these reasons make that they fall back to using one-sided directive advice (paternalistic decision making) in daily clinical practice (Driever et al., 2020). The pulmonologists and specialized respiratory nurses using the COPDnet Integral Diagnostic Trajectory intervention were already familiar with motivational interviewing and shared decision making in their daily practice. In fact, the discussion of the PPC is motivational interviewing with the aid of the graphical chart (Vercoulen, 2012). Yet in two third of the patients more than two consultation days were needed to complete the individual care plan based on the patients’ goals. Although, time-management wise two consults would be preferable, the pulmonologist and specialized respiratory nurse were flexible in making an extra appointment when needed. The process of the patient was leading in this.

In the current study we found significant improvements in both patient groups, and in two third of the patients we observed a clinically relevant increase in activation after two or three visits. However, one-third of the patients reported low activation levels after two or three visits, patients are in other words still passive and report to lack knowledge or confidence. In these patients the motivational interviewing techniques and shared decision-making processes did not have the desired effect on improving their activation level. Additionally, when focusing on the group with low activation levels at baseline, half did move to the activated levels (level 3–4) after the Integral Diagnostic Trajectory. These patients had initially significantly but not clinically relevant higher scores, but were comparable on age, sex, lung function, number of severe problems, education level, and employment status (data not shown). This is in line with the overall results that showed that baseline activation scores and change scores could only be predicted to a maximum of 24%. On item level significantly more patients in the group who progressed from the low activated to the activated levels reported at baseline to agree with the item “I am confident I can maintain lifestyle changes, like eating right and exercising, even during times of stress” as compared the group of patients who maintained low activated. And a discriminant analysis revealed that 72% of the patients could be correctly classified in “staying low activated” or “become activated” in the group with level 1 or level 2 at baseline, based on the items “I am confident I can maintain lifestyle changes, like eating right and exercising, even during times of stress” and “I have been able to maintain (keep up with) lifestyle changes, like eating right or exercising” (data not shown). Underlining the influence of previous experiences and belief in oneself in the possibility of changing patient activation. Positive experiences can be used as examples, whereas negative experiences need a different approach.

Other studies did not find any prognostic and socio-economic factors (Turner et al., 2014) or could predict activation scores for 16% (Bos-Touwen et al., 2015) to 45%, (Wetzstein et al., 2020) leaving much room for other still unidentified factors. Possible explanation could be the variations in preference between patients and even within patients, depending on the kind of decision that has to be made, for paternalistic or shared decision making (Zizzo et al., 2017). Deen et al. (2011) found that patients who preferred an active role also had significantly higher PAM scores compared to those who preferred a collaborative or passive role. Underlining the importance of getting the patient informed, develop skills and improve their self-esteem.

Moreover, most of the patients want to gain knowledge about the disease and possible treatment options. It is important to acknowledge these personal differences and to check during the consultation what the preferences of the patient are. More research is needed to get insight in why patients do not show improvement on the PAM after an intervention aiming at patient activation, and how to identify these patients at front. A result might be that shared decision making and coming to an individual care plan is not (yet) feasible for this group and further investments in their activation needs to be made.

### Strengths and limitations

Our study sample comprised of an unbiased, real-world population referred to the pulmonologist.

There are several limitations in the present study. First, the PAM score after the intervention was not collected from all patients. However, when comparing the proportions of patients of all patients who returned the baseline PAM with the proportions of patients who also returned the second PAM, the percentages of patients per activation level at baseline are comparable divided. Secondly, patients were followed only until directly after the intervention, it would be of interest to study whether these improvements in activation level are fostered and lead to changes in self-management and health status in the long run. Third, we did not include a control group so we could not confirm that the changes in patient activation are the result of the Integral Diagnostic Trajectory-intervention or whether these improvements also would have been accomplished by separate consultations with the pulmonologist and specialized pulmonary nurse.

The present study showed that a low level of activation for self-management is a common feature in patients with asthma and COPD referred for a first-ever outpatient hospital-based consultation. In addition, patients with asthma or COPD who participated in a diagnostic care pathway aiming to get a comprehensive understanding of the patients’ health status and to enhance the activation for self-management, higher levels of patient activation were achieved with a relatively short and seemingly simple intervention.

## Data availability statement

The raw data supporting the conclusions of this article will be made available by the authors, without undue reservation.

## Ethics statement

The studies involving human participants were reviewed and approved by The Research Ethics Committee of the Radboud University Medical Centre. Written informed consent for participation was not required for this study in accordance with the national legislation and the institutional requirements.

## Author contributions

JP conceived the study, performed the statistical analyses, and participated in the drafting of the manuscript. JA and EK conceived the study, extracted the data from the medical records, aided in interpreting the results, and participated in the drafting of the manuscript. JV aided in interpreting the results and participated in the drafting of the manuscript. HAH, HWH, MS, and BB reviewed and edited the manuscript. A‘tH was in charge of overall direction and reviewed and edited the manuscript. All authors provided critical feedback and helped to shape the research, analysis and manuscript, and approved the submitted version.

## References

[B1] Allegrante J. P. Wells M. T. Peterson J. C. (2019). Interventions to support behavioral self-management of chronic diseases. *Annu. Rev. Public Health* 40 127–146. 10.1146/annurev-publhealth-040218-044008 30601717 PMC6684026

[B2] Barker I. Steventon A. Williamson R. Deeny S. R. (2018). Self-management capability in patients with long-term conditions is associated with reduced healthcare utilisation across a whole health economy: Cross-sectional analysis of electronic health records. *BMJ Qual. Saf.* 27 989–999. 10.1136/bmjqs-2017-007635 30139822 PMC6288702

[B3] Beck A. T. Guth D. Steer R. A. Ball R. (1997). Screening for major depression disorders in medical inpatients with the beck depression inventory for primary care. *Behav. Res. Ther.* 35 785–791. 10.1016/S0005-7967(97)00025-9 9256522

[B4] Bergner M. Bobbitt R. A. Carter W. B. Gilson B. S. (1981). The sickness impact profile: Development and final revision of a health status measure. *Med. Care* 19 787–805. 10.1097/00005650-198108000-00001 7278416

[B5] Bloem A. E. M. Mostard R. L. M. Stoot N. Custers J. W. H. Vooijs M. Janssen D. J. A. (2022). Patient activation for self-management in patients with idiopathic pulmonary fibrosis or sarcoidosis. *Respiration* 101 76–83. 10.1159/000518216 34515234

[B6] Boer L. M. Daudey L. Peters J. B. Molema J. Prins J. B. Vercoulen J. H. (2014). Assessing the stages of the grieving process in chronic obstructive pulmonary disease (Copd): Validation of the acceptance of disease and impairments questionnaire (Adiq). *Int. J. Behav. Med.* 21 561–570. 10.1007/s12529-013-9312-3 23645551

[B7] Bos-Touwen I. Schuurmans M. Monninkhof E. M. Korpershoek Y. Spruit-Bentvelzen L. Ertugrul-Van Der Graaf I. (2015). Patient and disease characteristics associated with activation for self-management in patients with diabetes, chronic obstructive pulmonary disease, chronic heart failure and chronic renal disease: A cross-sectional survey study. *PLoS One* 10:e0126400. 10.1371/journal.pone.0126400 25950517 PMC4423990

[B8] Chang Y. Y. Dai Y. T. (2019). The efficacy of a flipping education program on improving self-management in patients with chronic obstructive pulmonary disease: A randomized controlled trial. *Int. J. Chron. Obstruct. Pulmon. Dis.* 14 1239–1250. 10.2147/COPD.S196592 31289439 PMC6565933

[B9] Collinsworth A. W. Brown R. M. James C. S. Stanford R. H. Alemayehu D. Priest E. L. (2018). The impact of patient education and shared decision making on hospital readmissions for Copd. *Int. J. Chron. Obstruct. Pulmon. Dis.* 13 1325–1332. 10.2147/COPD.S154414 29731620 PMC5927146

[B10] Deen D. Lu W. H. Rothstein D. Santana L. Gold M. R. (2011). Asking questions: The effect of a brief intervention in community health centers on patient activation. *Patient Educ. Couns.* 84 257–260. 10.1016/j.pec.2010.07.026 20800414

[B11] Diener E. Emmons R. A. Larsen R. J. Griffin S. (1985). The satisfaction with life scale. *J. Pers. Assess.* 49 71–75. 16367493 10.1207/s15327752jpa4901_13

[B12] Driever E. M. Stiggelbout A. M. Brand P. L. P. (2020). Shared decision making: Physicians’ preferred role, usual role and their perception of its key components. *Patient Educ. Couns.* 103 77–82. 10.1207/s15327752jpa4901_13 31431308

[B13] Effing T. W. Bourbeau J. Vercoulen J. Apter A. J. Coultas D. Meek P. (2012). Self-management programmes for copd: Moving forward. *Chron. Respir. Dis.* 9 27–35. 10.1177/1479972311433574 22308551

[B14] Effing T. W. Vercoulen J. H. Bourbeau J. Trappenburg J. Lenferink A. Cafarella P. (2016). Definition of a copd self-management intervention: International expert group consensus. *Eur. Respir. J.* 48 46–54. 10.1183/13993003.00025-2016 27076595

[B15] Eikelenboom N. Van Lieshout J. Jacobs A. Verhulst F. Lacroix J. Van Halteren A. (2016). Effectiveness of personalised support for self-management in primary care: A cluster randomised controlled trial. *Br. J. Gen. Pract.* 66 e354–e361. 10.3399/bjgp16X684985 27080318 PMC4838448

[B16] Elwyn G. Durand M. A. Song J. Aarts J. Barr P. J. Berger Z. (2017). A three-talk model for shared decision making: multistage consultation process. *BMJ* 359:j4891. 10.1136/bmj.j4891 29109079 PMC5683042

[B17] Greene J. Hibbard J. H. (2012). Why does patient activation matter? An examination of the relationships between patient activation and health-related outcomes. *J. Gen. Intern. Med.* 27 520–526. 10.1007/s11606-011-1931-2 22127797 PMC3326094

[B18] Hibbard J. H. Greene J. (2013). What the evidence shows about patient activation: Better health outcomes and care experiences; fewer data on costs. *Health Aff. (Millwood)* 32 207–214. 10.1377/hlthaff.2012.1061 23381511

[B19] Hibbard J. H. Mahoney E. R. Stock R. Tusler M. (2007). Do increases in patient activation result in improved self-management behaviors? *Health Serv. Res.* 42 1443–1463. 10.1111/j.1475-6773.2006.00669.x 17610432 PMC1955271

[B20] Hibbard J. H. Mahoney E. R. Stockard J. Tusler M. (2005). Development and testing of a short form of the patient activation measure. *Health Serv. Res.* 40 1918–1930. 10.1111/j.1475-6773.2005.00438.x 16336556 PMC1361231

[B21] Hibbard J. H. Stockard J. Mahoney E. R. Tusler M. (2004). Development of the patient activation measure (Pam): Conceptualizing and measuring activation in patients and consumers. *Health Serv. Res.* 39 1005–1026. 10.1111/j.1475-6773.2004.00269.x 15230939 PMC1361049

[B22] Humphries M. D. Welch P. Hasegawa J. Mell M. W. (2021). Correlation of patient activation measure level with patient characteristics and type of vascular disease. *Ann. Vasc. Surg.* 73 55–61. 10.1016/j.avsg.2020.11.019 33385528 PMC8882319

[B23] Janssen S. Spruit M. A. Antons J. C. Djamin R. S. Tjalma T. Veen T. (2020). Prevalence of non-pharmacological treatable traits in patients with asthma. *Eur. Resp. J.* 56:941. 10.5507/bp.2020.056 33325455

[B24] Juniper E. O’byrne P. Guyatt G. Ferrie P. King D. (1999). Development and validation of a questionnaire to measure asthma control. *Eur. Respir. J.* 14 902–907. 10.1034/j.1399-3003.1999.14d29.x 10573240

[B25] Koolen E. H. Van Den Borst B. De Man M. Antons J. C. Robberts B. Dekhuijzen P. N. R. (2020). The clinical effectiveness of the copdnet integrated care model. *Respir. Med.* 172:106152. 10.1016/j.rmed.2020.106152 32956973

[B26] Koolen E. H. Van Der Wees P. J. Westert G. P. Dekhuijzen R. Heijdra Y. F. Van ’T Hul A. J. (2018a). The copdnet integrated care model. *Int. J. Chron. Obstruct. Pulmon. Dis.* 13 2225–2235. 10.2147/COPD.S150820 30050295 PMC6056161

[B27] Koolen E. H. Van Der Wees P. J. Westert G. P. Dekhuijzen R. Heijdra Y. F. Van ’T Hul A. J. (2018b). Evaluation of the copdnet integrated care model in patients with copd: The study protocol. *Int. J. Chron. Obstruct. Pulmon. Dis.* 13 2237–2244. 10.2147/COPD.S153992 30050296 PMC6056168

[B28] Koolen E. H. Van ’T Hul A. J. Van Der Wees P. Dekhuijzen P. N. R. Heijdra Y. H. (2017). Activation for self-management in asthma or copd patients referred to a pulmonologist. *Eur. Respir. J.* 50:A3672. 10.1183/1393003.congress-2017.PA3672

[B29] Korpershoek Y. Bos-Touwen I. D. De Man-Van Ginkel J. M. Lammers J. W. Schuurmans M. J. Trappenburg J. (2016). Determinants of activation for self-management in patients with copd. *Int. J. Chron. Obstruct. Pulmon. Dis.* 11 1757–1766. 10.2147/COPD.S109016 27536087 PMC4976914

[B30] Koyama T. Nawa N. Itsui Y. Okada E. Fujiwara T. (2022). Facilitators and barriers to implementing shared decision making: A cross-sectional study of physicians in Japan. *Patient Educ. Couns.* 105 2546–2556. 10.1016/j.pec.2022.01.016 35184910

[B31] Mahler D. A. Wells C. K. (1988). Evaluation of clinical methods for rating dyspnea. *Chest* 93 580–586. 10.1378/chest.93.3.580 3342669

[B32] Maille A. R. Koning C. J. Zwinderman A. H. Willems L. N. Dijkman J. H. Kaptein A. A. (1997). The development of the ‘Quality-of-life for Respiratory Illness Questionnaire (Qol-Riq)’: A disease-specific quality-of-life questionnaire for patients with mild to moderate chronic non-specific lung disease. *Respir. Med.* 91 297–309. 10.1016/s0954-6111(97)90034-2 9176649

[B33] Miller W. R. Rollnick S. (2002). *Principles of motivational interviewing: Preparing people for change.* New York, NY: Guilford Press.

[B34] Nguyen H. Q. Gill D. P. Wolpin S. Steele B. G. Benditt J. O. (2009). Pilot study of a cell phone-based exercise persistence intervention post-rehabilitation for Copd. *Int. J. Chron. Obstruct. Pulmon. Dis.* 4 301–313. 10.2147/copd.s6643 19750190 PMC2740952

[B35] North M. Bourne S. Green B. Chauhan A. J. Brown T. Winter J. (2020). A randomised controlled feasibility trial of E-health application supported care vs usual care after exacerbation of copd: The rescue trial. *NPJ Digit. Med.* 3:145. 10.1038/s41746-020-00347-7 33145441 PMC7603326

[B36] Peters J. B. Daudey L. Heijdra Y. F. Molema J. Dekhuijzen P. N. Vercoulen J. H. (2009). Development of a battery of instruments for detailed measurement of health status in patients with copd in routine care: The Nijmegen clinical screening instrument. *Qual. Life Res.* 18 901–912. 10.1007/s11136-009-9502-2 19543807 PMC2724638

[B37] Pinnock H. Epiphaniou E. Pearce G. Parke H. Greenhalgh T. Sheikh A. (2015). Implementing supported self-management for asthma: A systematic review and suggested hierarchy of evidence of implementation studies. *BMC Med.* 13:127. 10.1186/s12916-015-0361-0 26032941 PMC4465463

[B38] Rademakers J. Nijman J. Van Der Hoek L. Heijmans M. Rijken M. (2012). Measuring patient activation in The Netherlands: Translation and validation of the American short form patient activation measure (Pam13). *BMC Public Health* 12:577. 10.1186/1471-2458-12-577 22849664 PMC3490810

[B39] Rygg L. O. Rise M. B. Gronning K. Steinsbekk A. (2012). Efficacy of ongoing group based diabetes self-management education for patients with type 2 diabetes mellitus. A randomised controlled trial. *Patient Educ. Couns.* 86 98–105. 10.1016/j.pec.2011.04.008 21592715

[B40] Titova E. Salvesen O. Bentsen S. B. Sunde S. Steinshamn S. Henriksen A. H. (2017). Does an integrated care intervention for copd patients have long-term effects on quality of life and patient activation? A Prospective, open, controlled single-center intervention study. *PLoS One* 12:e0167887. 10.1371/journal.pone.0167887 28060921 PMC5218408

[B41] Turner A. Anderson J. Wallace L. Kennedy-Williams P. (2014). Evaluation of a self-management programme for patients with chronic obstructive pulmonary disease. *Chron. Respir. Dis.* 11 163–172. 10.1177/1479972314539979 24980127

[B42] van ‘t Hul A. J. Koolen E. H. Antons J. C. De Man M. Djamin R. S. Johannes C. C. M. (2020). Treatable traits qualifying for nonpharmacological interventions in copd patients upon first referral to a pulmonologist: The copd straitosphere. *Eur. Resp. J. Open Res.* 6:00438. 10.1183/23120541.00438-2020 33263050 PMC7682701

[B43] van der Molen T. Willemse B. W. Schokker S. Ten Hacken N. H. Postma D. S. Juniper E. F. (2003). Development, validity and responsiveness of the clinical copd questionnaire. *Health Qual. Life Outcomes* 1:13. 10.1186/1477-7525-1-13 12773199 PMC156640

[B44] Vercoulen J. H. (2012). A simple method to enable patient-tailored treatment and to motivate the patient to change behaviour. *Chron. Respir. Dis.* 9 259–268. 10.1177/1479972312459974 23129804

[B45] Vercoulen J. H. Daudey L. Molema J. Vos P. J. Peters J. B. Top M. (2008). An integral assessment framework of health status in chronic obstructive pulmonary disease (copd). *Int. J. Behav. Med.* 15 263–279. 10.1080/10705500802365474 19005926

[B46] Vercoulen J. H. Swanink C. M. Fennis J. F. Galama J. M. Van Der Meer J. W. Bleijenberg G. (1994). Dimensional assessment of chronic fatigue syndrome. *J. Psychosom. Res.* 38 383–392. 10.1016/0022-3999(94)90099-X 7965927

[B47] Verhage F. (1965). Intelligence and age in a dutch sample. *Hum. Dev.* 8 238–245.

[B48] Wetzstein M. M. Shanta L. L. Chlan L. L. (2020). Patient activation among community-dwelling persons living with chronic obstructive pulmonary disease. *Nurs. Res.* 69 347–357. 10.1159/000270308 32404586

[B49] Yadav U. N. Hosseinzadeh H. Baral K. P. (2018). Self-management and patient activation in copd patients: An evidence summary of randomized controlled trials. *Clin. Epidemiol. Glob. Health* 6:7. 10.1136/bmjopen-2021-054659 34937723 PMC8705223

[B50] Zizzo N. Bell E. Lafontaine A. L. Racine E. (2017). Examining chronic care patient preferences for involvement in health-care decision making: The case of Parkinson’s disease patients in a patient-centred clinic. *Health Expect.* 20 655–664. 10.1111/hex.12497 27624704 PMC5513015

